# Failure Rate of Direct High-Viscosity Glass-Ionomer *Versus* Hybrid Resin Composite Restorations in Posterior Permanent Teeth - a Systematic Review

**DOI:** 10.2174/1874210601509010438

**Published:** 2015-12-22

**Authors:** Steffen Mickenautsch, Veerasamy Yengopal

**Affiliations:** SYSTEM Initiative/Department of Community Dentistry, Faculty of Health Sciences, University of the Witwatersrand - 7 York Rd., Parktown/Johannesburg 2193, South Africa

**Keywords:** Failure rate, high-viscosity glass-ionomers, posterior permanent teeth, resin composite, systematic review, tooth restorations

## Abstract

**Purpose:**

Traditionally, resin composite restorations are claimed by reviews of the dental literature as being superior to glass-ionomer fillings in terms of restoration failures in posterior permanent teeth. The aim of this systematic review is to answer the clinical question, whether conventional high-viscosity glass-ionomer restorations, in patients with single and/or multi-surface cavities in posterior permanent teeth, have indeed a higher failure rate than direct hybrid resin composite restorations.

**Methods:**

Eight databases were searched until December 02, 2013. Trials were assessed for bias risks, in-between datasets heterogeneity and statistical sample size power. Effects sizes were computed and statistically compared. A total of 55 citations were identified through systematic literature search. From these, 46 were excluded. No trials related to high-viscosity glass-ionomers *versus* resin composite restorations for direct head-to-head comparison were found. Three trials related to high-viscosity glass-ionomers *versus* amalgam and three trials related to resin composite *versus* amalgam restorations could be included for adjusted indirect comparison, only.

**Results:**

The available evidence suggests no difference in the failure rates between both types of restoration beyond the play of chance, is limited by lack of head-to-head comparisons and an insufficient number of trials, as well as by high bias and in-between-dataset heterogeneity risk. The current clinical evidence needs to be regarded as too poor in order to justify superiority claims regarding the failure rates of both restoration types. Sufficiently large-sized, parallel-group, randomised control trials with high internal validity are needed, in order to justify any clinically meaningful judgment to this topic.

## INTRODUCTION

In recent years, the use of resin composites for the placement of posterior permanent tooth restorations has increased, particularly because of its better esthetic properties and the general concerns about the limitations of amalgam [1, 2]. Notwithstanding, a systematic review by Antony *et al.* (2008) concluded that composite resin fillings show a shorter longevity than amalgam fillings [3].

In contrast to resin composite, conventional glass-ionomer cements (C-GIC), defined as chemically curing materials without a resin component, have traditionally been considered as inferior restorative materials [4-7]. Downer *et al.* (1999) [4], Hickel *et al.* (2000) [5], Hickel and Manhart (2001) [6] and Manhart *et al.* (2004) [7] have published influential and highly cited reviews of the dental literature with relevance to the longevity of various restorative materials, including C-GIC. The findings of these reviews, based on mainly uncontrolled longitudinal studies and laboratory investigations, conclude that C-GICs are less durable, with a far higher failure rate, than resin composite when placed as posterior occlusal or approximal restorations [4-7]. For these reasons C-GIC were regarded as lacking adequate mechanical properties for general use as definitive restorations in stress-bearing posterior teeth.

Over the last twenty years, the term ‘high-viscosity’ or ‘high-viscous’ C-GICs (HVGIC) has emerged within the scientific dental literature. HVGICs appear distinct from other (low) viscosity C-GICs (including metal-reinforced C-GICs/Cermets) in comparison of their survival rate to that of conventional amalgam restorations. The results of a meta-analysis by Frencken *et al.* (2004) found a survival rate for HVGIC (Fuji IX; Ketac Molar) similar to that of amalgam but showed significantly lower survival rates for “low-viscosity” GICs (Chelon Silver/Cermet; Chem Fil; Fuji II) than for amalgam [8]. Subsequent systematic review evidence from clinical randomised control trials (RCTs) has confirmed the earlier meta-analysis results [9, 10]. In particular for direct HVGIC restorations placed in the permanent dentition, the clinical evidence shows: (i) no difference between single-surface HVGIC and conventional amalgam restorations in their failure rates after six years; (ii) no difference in the failure rates of multiple-surface HVGIC and conventional amalgam restorations after four years [9, 10]. 

Despite many published reviews of the dental literature [4-7], no systematic review evidence based on the comparative effect estimates concerning the clinical failure rate of direct tooth restorations placed in posterior permanent teeth using HVGIC, with resin composite as control intervention, is available. Therefore, the aim of this systematic review was to answer the question as to whether conventional high-viscosity glass-ionomer restorations, in patients with single and/or multi-surface cavities in posterior permanent teeth, have indeed a higher failure rate than direct hybrid resin composite restorations.****

## MATERIALS AND METHODOLOGY

The protocol of this systematic review has been registered with the International Prospective Register of Systematic Reviews (PROSPERO / Nr: CRD42013006487) and was published in an open access journal [11].

### Systematic Literature Search

Electronic international databases: PubMed/Medline and CENTRAL (Cochrane Library); databases of open access journals: Directory of Open Access Journals (DOAJ) and Biomed Central; regional databases: IndMed and Sabinet, as well as databases for grey-literature: OpenSIGLE and GoogleScholar were searched by both authors independently. The details of the search strategies, including the strings of search terms (containing MeSH and text search terms) together with Boolean operators, per databases are presented in Additional file **S1/Section 1**. All databases were searched until 02.12.2013, the cut-off date. Citations were eligible for possible inclusion if in line with the following inclusion criteria:

- Full report of a prospective clinical control trial
- Clearly reported parallel group design with dichotomous outcome measure
- Outcome measure: restoration failure due to fracture, wear, and secondary caries and/or retention loss
- Intervention in load bearing cavities of permanent posterior teeth
- Follow-up period: 1 year or longer with longest comparable result per study
- Trials comparing:
  - Resin composite *versus* amalgam
  - HVGIC *versus* amalgam
  - HVGIC *versus* resin composite

Trial participants comprised all patients of any age, gender or place of origin with suitable tooth cavities in posterior permanent teeth. 

Articles were further excluded according to the criteria:

- Tunnel restoration or preventive resin restoration (sealed restoration) as intervention type
- Cermet or low-viscosity glass-ionomer cements as test intervention
- Chemically curing composite resin or non-hybrid resin composite as control intervention
- Low-copper amalgam alloys as control intervention
- Resin composite and/or amalgam restorations placed with GIC liner or base
- No computable data (*n* = number of failures; *N* = total number of evaluated units, per intervention group) reported
- Tooth cavities on anterior teeth, Class V cavities or cavities on root/cervical tooth surfaces
- Tooth cavities extending into enamel only
- For possible network-meta analysis or indirect treatment comparison (ITC), only datasets that matched in regard to restored cavity type and follow-up period, as well as with low statistical heterogeneity (Cochrane’s Q-test/*p* > 0.10/ *I^2^* < 45%) were included.
- Articles that could not be traced in full copy were also excluded.

Two reviewers (SM and VY) scanned titles and abstracts of identified citations from data sources in duplication. Articles with suitable titles but lacking listed abstracts were retrieved in full copy. All included articles were judged separately by the authors: for possible exclusion, with reason, or for acceptance, in line with the exclusion criteria. Disagreements between authors were resolved through discussion and consensus. 

### Assessment of Internal Validity/Bias Risk

Verbatim quotes relevant to selection-, performance- and detection bias risk were extracted from all accepted trial reports. Assessment of the risk of selection bias and detection/performance bias was based on these, using criteria [12], shown in Additional file **S1/Section 2**. 

In order to assess attrition bias risk, worst- and best-case scenarios were assumed. These were calculated in cases were the number of lost trial participants per intervention group was reported in the trial reports. The results were then compared to the intervention outcomes computed for participants available to follow-up. On this basis conclusions concerning attrition bias risk were drawn: high risk of attrition bias was assumed if the computed outcomes between worst- and best-case scenario and the intervention outcomes computed for participants available to follow-up differed significantly. Where the number of lost trial participants per intervention group was not reported, a high risk of attrition bias was assumed by default. 

The worst-case scenario was calculated by adding the number of lost-to-follow-up participants in the test group to the failures of that group and adding the number of lost-to-follow-up participants in the control group to the successes of that group. The best-case scenario was calculated by adding the number of lost-to-follow-up participants in the test group to the successes of that group and adding the number of lost-to-follow-up participants in the control group to the failures of that group. Intervention outcomes were computed for assessment of attrition bias as Relative risks (RR) with 95% Confidence intervals (CI). The two reviewers conducted the assessment separately. Disagreements were resolved through discussion and consensus. 

In order to assess the risk of publication bias, the following trial registers and electronic sources were searched for abstracts and/or titles of trials in line with trial selection criteria as per sections: Australian New Zealand Clinical Trials Registry; Clinical Trials US; EU Clinical Trials Register; *meta*Register of Controlled Trials (*m*RCT); South African National Clinical Trials Register; WHO Clinical Trials; IADR abstracts; International Poster Journal of Dentistry and Oral Medicine. Comparison of the number of identified unpublished with the number of published (accepted) trials was used to assess the possible extent of potential publication bias risk.

Graphical and statistical assessments of publication bias risk were planned if the number of extracted datasets exceeded ten.

### Data Extraction and Statistical Analysis

Acceptable outcome measures were the number of restoration failures (*n*) from the total number of evaluated units (*N*) for dichotomous data. Restoration failures were defined as: fractured restorations; secondary caries on restoration margins; partial or total loss of restoration retention; excessive wear of the restoration material. Only primary outcomes with either the patient or the tooth as unit of analysis were accepted. For each extracted dataset of *n / N* for the test- and control groups, the Odds ratio (OR) with 95% Confidence intervals (CI) and significance levels (*p*-values) were computed, using the statistical software programme Rev Man 4.2. The 5% significance level was used. In addition, each accepted dataset was assessed as to whether it contained the minimum sample size needed in order to have >80% power for detecting an effect size difference of 10% between directly compared interventions. (Additional file **S2/Section 6**). The effect size difference was chosen in keeping with past considerations for sample size calculation [13]. 

Both authors extracted data from accepted trials independently, without being blinded to authors, institutions, journal names and trial results. Disagreements between authors concerning extracted data were solved through discussion and consensus. All extracted data were entered in specifically designed MS Excel data sheets. 

### Assessment and Investigation of In-Between-Dataset Heterogeneity

To fulfill the criteria of clinical and methodological homogeneity, datasets from trials should not have differed with regard to the following minimum set of characteristics: outcome measure; control intervention; cavity type restored; assessment method and length of follow-up period. If in-between-dataset differences were identified, heterogeneity was assumed and the datasets were excluded from analysis.

The *I^2^*– test with 95% CI, as well as Cochrane’s Q-test, was used to determine whether any statistical heterogeneity might exist between datasets. Thresholds for *I^2 ^*point estimates (in %) and their upper confidence values were used in order to interpret the test results: 0-40% = might not be important; 30-60% = may represent moderate heterogeneity; 50-90% = may represent substantial heterogeneity; 75-100% = considerable heterogeneity [14].

Across different trials, only datasets that showed lack of statistical in-between heterogeneity were considered eligible for indirect comparison. The cut-off point for statistical heterogeneity was set at *I^2^* > 40% and Cochrane’s Q-test/*p*-value < 0.10. However, datasets in compliance with these set test criteria were not assumed to be free of in-between heterogeneity, particularly due to the weak statistical power of both tests when the number of datasets are low [15], but were further investigated in the form of trial characteristics comparison tables. 

### Types of Comparisons Between Test- and Control Groups

#### Direct Comparisons

Direct comparisons were considered to be comparisons between intervention groups within a controlled clinical trial setting [16]. The systematic literature search of this review aimed to identify clinical trials that directly compared HVGIC with resin composite restorations, in line with the stated inclusion/exclusion criteria.

#### Adjusted Indirect Comparisons

It has been reported that only a few clinical trials that have directly compared HVGIC with resin composite restorations have yet been conducted [17]. As it was possible that any existing clinical trials may not have complied with the stated inclusion/exclusion criteria of this review, the use of adjusted indirect comparison was considered. Adjusted indirect comparison between intervention groups may preserve the strength of an RCT, if adjusted according to the results of their direct comparison with a common control [18]. Therefore, the systematic literature search was extended to identification of clinical trials that: (i) compare HVGIC with amalgam restorations and (ii) compare resin composite with amalgam restorations. Consequently, this systematic review would also statistically compare the clinical results of HVGIC *versus* amalgam trials with the clinical results of the resin composite *versus* amalgam trials through use of Indirect Treatment Comparison (ITC) or, if possible, network meta-analysis [19, 20].

For ITC, the statistical methodology given by Bucher *et al.* (1997) was applied [21, 22]. The results were confirmed through use of the ITC software developed by the Canadian Agency for Drugs and Technologies in Health (2009) [23]. All indirect comparison results were reported as Odds ratios (OR with 95% CI). 

## RESULTS

### Systematic Literature Search and Data Selection

**Fig. (1):**
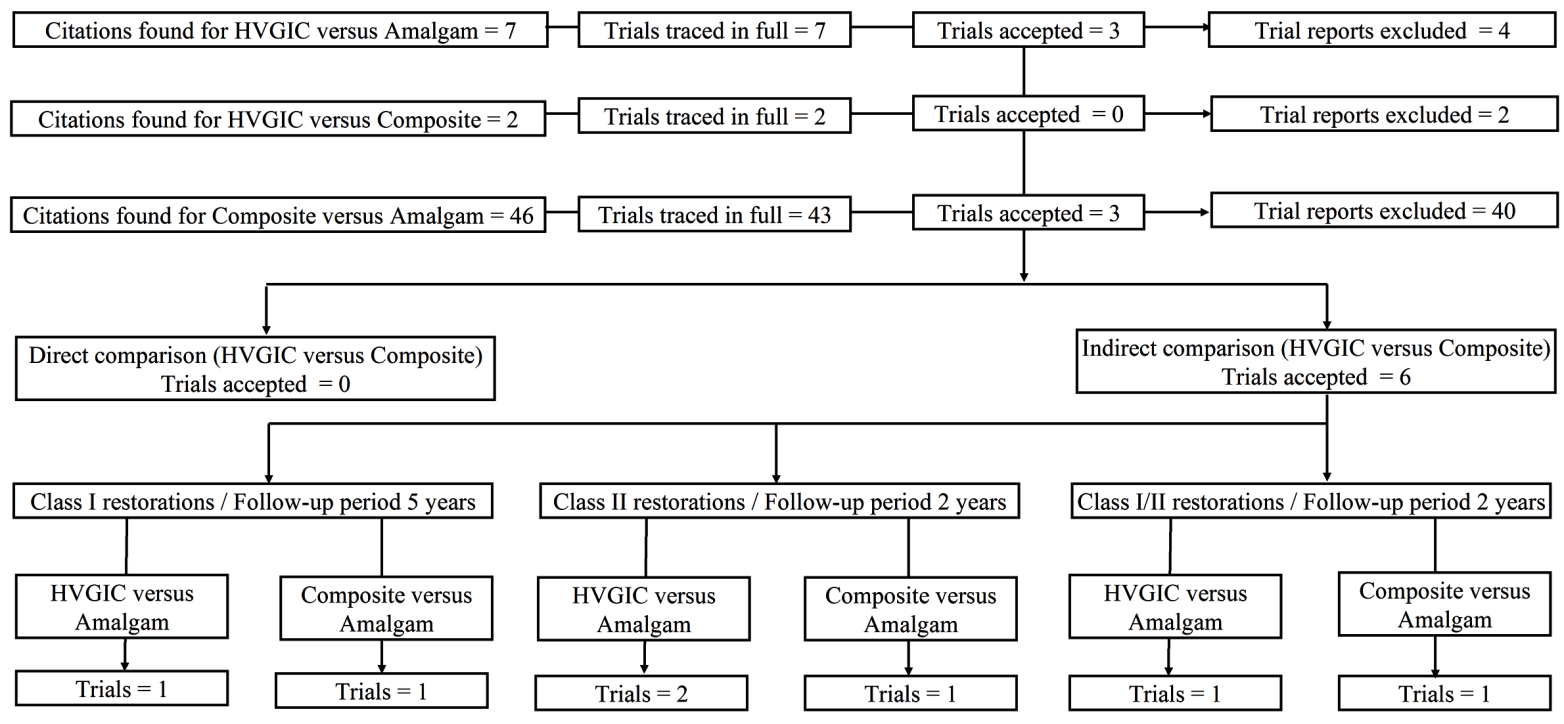
Flow diagram of trial selection.

(Fig. **1**) provides information on the number of articles identified. From the generated citations, 55 clinical trials were provisionally included. Of these, three could not be traced in full [24-26].

Thus 52 trials were included for further review: 7 trials were related to HVGIC *versus* amalgam restorations, 2 trials were related to HVGIC *versus* resin composite restorations and 43 trials were related to composite *versus* amalgam restorations. 

From these trials, a total of 46 were excluded. Reasons for exclusion are listed in Additional file **S2/Section 1**.

Six trials were accepted for data extraction: three related to HVGIC *versus* amalgam restorations [27-29] and three to resin composite *versus* amalgam restorations [2, 30, 31]. Details of all accepted trials are presented in Additional file **S2/Section 3**. 

### Assessment of Internal Trial Validity/Bias Risk

Assessment of selection- and performance-/detection bias risk was based on verbatim quotes extracted from all six accepted trials (Additional file **S2/Section 5**). Details of the assessment results are presented in Table **1**.

The risk of selection- and performance-/detection bias was judged to be high for all trials. Only two trials reported adequate methods of random sequence generation and concealment of the random sequence in order to prevent direct observation (selection bias risk) [29, 30]. None of the trials reported adequate methods of patient and clinical operator masking as to the type of treatment rendered (performance bias risk) and only two trials [28, 30] reported adequate methods for masking evaluators during the trial assessment period (detection bias risk). 

Only datasets that were judged sufficiently homogeneous in line with the trial inclusion criteria were quantitatively assessed for attrition bias risk. The results are presented in Table **2** and indicate high bias risk for all except two datasets (CA01 and CA04) from two accepted trials [2, 31]. 

As the number of accepted datasets was < 10, no statistical and graphical assessment of publication bias risk was conducted. The search results from international and regional clinical trial registers and presented congress abstracts (Additional file **S2/Section 8**) identified six further studies that were presented from 2005 – 2013 at dental congresses. Of these, five studies appeared to remain unpublished to date; while one study was only partially published in the format of a case report in a regional dental magazine [32]. All six studies appeared to be of split-mouth study design and thus would not have met the inclusion criteria of this review (Additional file **S3**).

### Extracted Data and Analysis

From the six accepted clinical trials, 21 datasets were extracted. In addition, the review authors computed three further datasets from the information given in one trial report (Additional file **S2/Section 3 and 4**) [2]. No datasets for direct comparison between HVGIC and resin composite restorations in line with the set review inclusion criteria were identified. 

Of the total 24 datasets nine datasets from three trials comparing composite resin *versus* amalgam restorations could be matched with nine datasets from three trials comparing HVGIC *versus* amalgam restorations, according to the minimum set of characteristics for clinical/methodological homogeneity: outcome measure; control intervention; cavity type restored; assessment method and length of follow-up period (Table **3**). Each of the nine pairs was investigated for statistical in-between-datasets heterogeneity (*I^2^* > 40%; Cochrane’s Q-test/*p* < 0.10). Accordingly, five pairs were excluded and four pairs accepted for ITC (Table **4**). All four pairs of accepted datasets appeared to be statistically homogeneous (*I^2^* range: 0 - 39%; Cochrane’s Q-test/*p*-value range: 0.20 – 0.96) and comprised:

- Two pairs of datasets, CA01/GA02 [27, 31] and CA01/GA10 [28, 31], regarding tooth restorations placed in Class II cavities, after follow-up period of 24 months;
- One pair of datasets, CA02/GA07 [28, 30], regarding tooth restorations placed in Class I cavities, after follow-up period of 60 months;
- One pair of datasets, CA04/GA17 [2, 29], regarding tooth restorations placed in Class I/II cavities, after follow-up period of 24 months.

The characteristics of the datasets in the four pairs are listed in Additional file **S2/Section 7**. The type of information presented in the trial report about trial characteristics varied greatly and thus made further in-depth comparison within each dataset pair impossible. However, from the comparison tables it can be discerned that patients within the dataset pairs CA01/GA02 [27, 31] and CA01/GA10 [28, 31] differed in age, while all other dataset pairs indicated similar age groups (Additional file **S2/Section 7**). No meta-analysis was conducted, due to lack of sufficient homogeneous data.

Assessment of all accepted datasets concerning the minimum sample size needed in order to have >80% power for detecting an effect size difference of 10% between directly compared interventions showed that the sample size of the datasets included in the two ITCs concerning Class II restorations after 24 months were too small. However, the sample sizes in all datasets for the ITCs concerning Class I restorations after 60 months and Class I/II restorations after 24 months appeared sufficient (Additional file **S2/Section 6**).

The results of the ITC analysis are presented in Table **4**. For the two ITCs concerning Class II restorations after 24 months, which involve zero cell counts, a fixed count of 0.5 was added to all cells of the treatment-by-outcome contingency table to avoid division by zero in the calculation of the Odds ratio. 

The results of all four ITCs show no statistically significant difference between the failure rate of load-bearing HVGIC and resin composite restorations when placed in posterior permanent teeth, after 24 and 60-month follow-up.

## DISCUSSION

### Limitations of The Systematic Review Method

The aim of this systematic review was to answer the question as to whether conventional high-viscosity glass-ionomer restorations, in patients with single and/or multi-surface cavities in posterior permanent teeth, have indeed a higher failure rate than direct hybrid resin composite restorations. Despite the applied systematic literature search, no trials directly comparing HVGIC *versus* resin composite restorations could be identified that complied with the adopted set of inclusion/exclusion criteria. No search of Chinese and Latin American databases was included in this review and this may have contributed artificially to the observed paucity of suitable trials. However, it has been suggested that exclusion of non-English trial reports has little effect on the overall conclusions of systematic reviews [33, 34] and this appeared to have been confirmed by the results of a further systematic review by the authors to the topic of HVGIC *versus* amalgam tooth restorations [10]. For that reason, and although the omission of searching Chinese and Latin American databases may have limited the conclusion of this systematic review in principle, this limitation is assumed to have had no significant influence on the presented review conclusions. 

Notwithstanding the lack of trials directly comparing HVGIC *versus* resin composite restorations, several trials comparing HVGIC *versus* amalgam and resin composite *versus* amalgam restorations could be identified. This allowed adjusted indirect comparison of the failure rates between HVGIC and composite resin, using the accepted ITC method by Bucher *et al.* (1997) [21]. However, the application of adjusted indirect comparison, as opposed to direct comparison of competing clinical interventions through RCTs, may have limited the precision of the presented results by generating wider confidence intervals [16]. Accordingly, adjusted indirect comparison results are less likely to be statistically significant (*p* > 0.05) than results from direct comparisons in RCTs. However, adjusted indirect comparison partially preserves the rigor of RCTs by considering the direct comparisons of both treatments of interest with the same controls [16]. Therefore, adjusted indirect comparison does not share the severe limitations of naïve-indirect comparisons. 

One further potential limitation of indirect comparisons is related to the high risks of heterogeneity between trial characteristics of the compared groups. Although, in this systematic review great care was taken to only admit datasets for comparison that showed small risk of statistical heterogeneity (Table **3**) and were sufficiently homogeneous in terms of outcome measure; control intervention; cavity type restored; assessment method and length of follow-up period, it was not possible to eliminate the risks of heterogeneity between the available datasets, completely. 

### Systematic Literature Search

The number of excluded studies, particularly for resin composite *versus* amalgam, was high (Additional file **S2/Section 1**). Most trial reports could not be accepted because they investigated older generations of restorative materials, such as chemically curing composite or low copper amalgam, that differed from those in current use and would have introduced further levels of clinical in-between-trial heterogeneity to the latter accepted RCTs. 

Care was further taken not to include trials with split-mouth study design. Thus three trials comparing HVGIC *versus* amalgam restorations were excluded (Additional file S2/Section 1). Characteristics, such as higher risk for cavities and poorer oral hygiene and dietary behaviour in patients with at least two cavities, as opposed to patients with only one cavity in trials with parallel-group design, would have introduced clinical in-between-trial heterogeneity that needed to be avoided during ITC analysis. Such rather strict approach in trial selection according to aspects of lowest bias risk and highest achievable precision was needed, owing to the paucity of direct comparison/RCT in the current literature and in order to derive the most reliable and valid results through the more problematic adjusted indirect comparison method, instead.

Two trials investigating direct comparison of HVGIC *versus* resin composite restorations were identified through the systematic literature search [17, 32]. However, both trials were excluded. The trial by Diem *et al.* (2013) included composite restorations with HVGIC base, only, that would have introduced clinical HVGIC characteristics into the composite treatment group [17]. The trial by Gurgan *et al.* (2013) was only partially reported in a dental magazine at search cut-off date and, thus was unsuitable for further review [32]. 

### Assessment of Internal Trial Validity/Bias Risk

All of the accepted trials appear limited by risk of either selection- and/or detection-/performance bias. 

Only two trials reported on the use of an adequate randomisation method [29, 30]. Nonetheless, all trials failed to report not only on evidence of successful sequence allocation and allocation concealment results, but also on necessary details about how sequence allocation and allocation concealment were attempted (Table **1**) [12]. None of the trials, therefore, provide any guarantee that each patient had an equal chance of being allocated to either treatment group. 

From the onset, in all trials successful blinding or masking appeared not to have been possible, owing to the obvious differences in clinical appearance between the compared materials. For that reason, the allocation to either treatment group was visible to patients and operators. Only two trials reported adequate methods of masking/blinding of the trial evaluators [28, 30]. However, no evidence was reported as to whether such methods were indeed successful. Against this background, the danger of detection-/performance bias may have affected the trial results. Potential knowledge of superiority claims prior to the trial may thus have led evaluators to apply different rigor in their assessments of the different treatment groups. 

On the basis of the results of the quantitative risk assessment (Table **2**), the risk for attrition bias may be regarded as high in all compared datasets, but particularly for the comparison of Class I tooth restorations after a 60-month period (DS CA02 *versus* GA07) [28, 30]. 

Owing to the low number of available datasets (*N * < 10) it was not possible to establish information concerning publication bias risk through graphical and statistical methods. However, the lack of listed trial protocols and relevant conference abstracts of unpublished trials suggests that the risk of publication bias to the reviewed topic may be low. 

### Statistical Analysis of Results

Based on the conducted ITC analysis, the 95% Confidence intervals of all four Odds ratios (ORs) included 1.00, thus indicating no statistically significant difference between HVGIC and resin composite restorations (Table **4**). The relatively large width of the confidence intervals may confirm previous observations [35] that adjusted indirect comparison gives lower precision than direct comparisons. Such lower precision may thus also be prevalent for ITC results for which a sufficiently high sample size in datasets was established (Additional file **S2/Section 6**).

### Recommendations for Further Research 

The available evidence suggests no difference in the failure rates between both types restoration beyond the play of chance, is limited by lack of head-to-head comparisons, an insufficient number of trials, as well as by high bias and in-between-dataset heterogeneity risk. Therefore the current evidence requires corroboration by large-sized, parallel-group, randomised control trials that compare HVGIC with resin composite restorations. Such future trials should avoid attrition bias risk through high loss-to-follow-up; include an investigation of performance/detection bias influence into its methodology, and test for selection bias risk, using the Berger-Exner test in order to quantitatively ascertain the level of adherence to the allocated random sequence throughout the trial [12]. 

## CONCLUSION

Superiority claims regarding the failure rates of direct hybrid resin composite restorations above that of conventional high-viscosity glass-ionomer restorations, in patients with single and/or multi-surface cavities in posterior permanent teeth, cannot be justified by the current poor clinical evidence to this topic. Direct head-to-head comparisons through clinical sufficiently large-sized parallel group randomised control trials with high internal validity are needed in order to justify any clinically meaningful judgement. 

## Figures and Tables

**Table 1.** Assessment of selection-, performance- and detection bias risk.

First author	Journal	Year	Vol.	First page	SB	PB	DB
Sachdeo [31]	EurJProsthodontRestDent	2004	12	15	0	0	0
Soncini [30]	JADA	2007	138	763	C	0	C
Bernado [2]	JADA	2007	138	775	0	0	0
Li [27]	PractClinMed	2005	6	105	0	0	0
Frencken [28]	JDR	2006	85	622	0	0	C
Estupiñán-Day [29]	PAHO-report/Ecuador	2006			C	0	0

SB = Selection bias; PB = Performance bias; DB = Detection bias; Vol. = Journal volume; EurJProsthodontRestDent = European Journal of Prosthodontic Dentistry; JADA = Journal of the American Dental Association; PractClinMed = Practice Clinical Medicine; JDR = Journal of Dental Research; PAHO = Pan-American Health Organisation.

**Table 2.** Assessment of attrition bias risk.

First author	Journal	Year	Vol.	Worst-case scenario																
				First page	DS	Test group			Amalgam group				LTF adjusted effect estimate				Original effect estimate			Bias risk
						LTF	N = BSL teeth	n+LTF	LTF	N+LT		n								
													RR	95% CI		p	RR	95% CI	p	
Sachdeo [31]	EurJProsthodontRestDent	2004	12	15	CA01	no LTF apparent during trial														No
Soncini [30]	JADA	2007	138	763	CA02	nil														Yes
Bernado [2]	JADA	2007	138	775	CA04	no LTF apparent during trial														No
Li [27]	PractClinMed	2005	6	105	GA02	nil														Yes
Frencken [28]	JDR	2006	85	622	GA07	18	52	21	10	33		10	1.33	0.72 - 2.46		0.36	0.20	0.06 - 0.66	0.008*	Yes
					GA10	127	288	135	105	218		5	20.44	8.52 - 49.03		<0.00001**	1.12	0.38 - 3.34	0.83	Yes
Estupiñán-Day [29]	PAHO-report/Panama	2006			GA17	144	769	166	118	677		8	18.27	9.03 - 36.86		<0.00001**	2.46	1.10 - 5.48	0.03**	No
First author	Journal	Year	Vol	Best-case scenario																
				First page	DS	Test group			Amalgam group				LTF adjusted effect estimate				Original effect estimate			Bias risk
						LTF	N+LTF teeth	n	LTF		N = BSL	n+LTF								
													RR		95% CI	p	RR	95% CI	p	
Sachdeo [31]	EurJProsthodontRestDent	2004	12	15	CA01	no LTF apparent during trial														No
Soncini [30]	JADA	2007	138	763	CA02	nil														Yes
Bernado [2]	JADA	2007	138	775	CA04	no LTF apparent during trial														No
Li [27]	PractClinMed	2005	6	105	GA02	nil														Yes
Frencken [28]	JDR	2006	85	622	GA07	18	52	3	10	33		20	0.10	0.03 - 0.30		<0.00001*	0.20	0.06 - 0.66	0.008*	No
					GA10	127	288	8	105	218		110	0.06	0.03 - 0.11		<0.00001*	1.12	0.38 - 3.34	0.83	Yes
Estupiñán-Day [29]	PAHO-report/Panama	2006			GA17	144	769	22	118	677		126	0.15	0.10 - 0.24		<0.00001*	2.46	1.10 - 5.48	0.03**	Yes

LTF = Number of restorations lost to follow-up; Vol. = Journal volume; DS = Dataset number; N = Number of restorations evaluated; BSL = Number of restorations at baseline; n = Number of failed restorations; RR = Risk ratio; CI = Confidence interval; EurJProsthodontRestDent = European Journal of Prosthodontic Dentistry; JADA = Journal of the American Dental Association; PractClinMed = Practice Clinical Medicine; JDR = Journal of Dental Research; PAHO = Pan-American Health Organisation; *Difference statistically significant in favour of test group; ** Difference statistically significant in favour of control group.

**Table 3.** Datasets matching according to cavity class and follow-up period.

Datasets	Cavity class	Follow Up (Mo)	Statistical in-between datasets heterogeneity				
			(Q) p-value	I^2^ (in %)	LCL	UCL	sHET
CA01 *versus* GA02	2	24	0.95	0	0	0	No
CA01 *versus* GA10	2	24	0.2	0	0	0	No
CA02 *versus* GA07	1	60	0.65	0	0	0	No
CA04 *versus* GA16	1+2	24	0.12	59	0	90	Yes
CA04 *versus* GA17	1+2	24	0.96	0	0	0	No
CA04 *versus* GA18	1+2	24	0.02	82	22	96	Yes
CA04 *versus* GA04+10	1+2	24	<0.0001	95	86	98	Yes
CA05 *versus* GA06+12	1+2	48	<0.0001	95	87	98	Yes
CA06 *versus* GA08	1+2	72	0.07	69	0	93	Yes

LCL = Lower 95% Confidence level; UCL = Upper 95% Confidence level; Mo = Months; sHET = Observed statistical heterogeneity.

**Table 4.** Indirect treatment comparison (ITC) results.

1. Single dataset results							
First author	Journal	Year	Vol.	First page	DS	OR	95%CI
(a) Composite resin *versus* amalgam restorations							
Sachdeo [31]	EurJProsthodont RestDent	2004	12	15	CA01	1.39	0.027 - 72.5
Soncini [30]	JADA	2007	138	763	CA02	1.95	1.26 - 3.01
Bernado [2]	JADA	2007	138	775	CA04	2.47	1.42 - 4.31
(b) HVGIC *versus* amalgam restorations							
Li [27]	PractClinMed	2005	6	105	GA02	1.52	0.80 - 2.90
Frencken [28]	JDR	2006	85	622	GA07	1.13	0.36 - 3.55
					GA10	0.13	0.03 - 0.53
Estupiñán-Day [29]	PAHO-report/Ecuador	2006			GA17	2.51	1.11 - 5.69
2. ITC results: HVGIC *versus* composite resin restorations							
DS-1	DS-2	OR	95% CI				
CA01	GA02	1.09	0.02 - 59.90				
CA01	GA10	0.09	0.001 - 6.08				
CA02	GA07	0.58	0.17 - 1.97				
CA04	GA17	1.02	0.38 - 2.73				

DS = Dataset number; Vol. = Journal volume; DS-1 = Dataset/Composite resin *versus* amalgam; DS-2 = Dataset/HVGIC *versus* amalgam; OR = Odds ratio; CI = Confidence interval; ln = Natural logarithm; SE = Standard error; EurJProsthodontRestDent = European Journal of Prosthodontic and Restorative Dentistry; JADA = Journal of the American Dental Association; PractClinMed = Practical Clinical Medicine (journal); JDR = Journal of Dental Research; PAHO = Pan-American Health Organisation; HVGIC = High-viscosity glass-ionomer cement.

## SUPPLEMENTARY MATERIAL

### Additional File S1

Systematic literature search / Internal validity criteria.

### Additional File S2

References, extracted data and information from accepted trials.

### Additional File S3

Congress abstracts.
