# Supplementary material for: Failure Rate of Direct High-Viscosity Glass-Ionomer Versus Hybrid Resin Composite Restorations in Posterior Permanent Teeth - a Systematic Review
Source: Open Dent J. 2015 Dec 22;9:438–48. doi: 10.2174/1874210601509010438 (PMC4768657; doi:10.2174/1874210601509010438)
Supplement: Supplementary file 1 [file TODENTJ-9-438_S1.pdf]

Open Access

Supplementary Material

Failure Rate of Direct High-Viscosity Glass-Ionomer *versus* Hybrid Composite Resin Restorations in Posterior Permanent Teeth - A Systematic Review

Steffen Mickenautsch* and Veerasamy Yengopal

**SYSTEM Initiative / Department of Community Dentistry, Faculty of Health Sciences, University of the Witwatersrand, 7 York Rd., Parktown/Johannesburg 2193, South Africa*

Additional file S1 – 1. Systematic literature search

1. Search strategy: electronic database search: Composite *versus* amalgam

Search term number	Electronic database	Number of Citations found
BMC search strategy: 02.12.2013<br>Online: http://www.biomedcentral.com/search/		
[1]	amalgam AND composite	37
**Articles included**		**0**
CENTRAL search strategy: 02.12.2013<br>Online: http://www.thecochranelibrary.com/view/0/index.html		
[1]	amalgam AND composite / Limit set: “Trials” , “Cochrane reviews”	188
**Articles included**		**15**
DOAJ search strategy: 02.12.2013<br>Online: http://www.doaj.org		
[1]	amalgam AND composite	37
**Articles included**		**0**
GoogleScholar search strategy: 02.12.2013<br>Online: http://scholar.google.co.za/		
[1]<br>[2]<br>[3]<br>[4]<br>[a]<br>[b]<br>[c]	amalgam AND composite<br>amalgam AND composite AND randomised control trial<br>amalgam AND composite AND randomised control trial AND longevity<br>amalgam AND composite AND randomised control trial AND longevity AND restoration<br>Custom range 2006 – 2013<br>[4a] + NOT review<br>[4b] + NOT book	About 37,300 results<br>About 2,620 results<br>About 999 results<br>About 718 results<br>About 560 results<br>About 523 results<br>About 268 results
**Articles included**		**0**
IndMed search strategy: 02.12.2013<br>Online: http://indmed.nic.in/		
[1]	amalgam AND composite	8
**Articles included**		**0**
OpenSIGLE search strategy: 02.12.2013<br>Online: http://opensigle.inist.fr/		
[1]	amalgam AND composite	1
**Articles included**		**0**
PubMed search strategy: 02.12.2013<br>Online: http://www.pubmed.org		
[1]<br>[2]<br><br>[a]	("Dental Amalgam"[Mesh]) AND "Composite Resins"[Mesh]<br>(("Dental Amalgam"[Mesh]) AND "Composite Resins"[Mesh]) AND "Dental Restoration, Permanent"[Mesh]<br>Limit set: “Clinical trial”	1518<br>1209<br><br>119
**Articles included**		**15**
SABINET search strategy: 02.12.13<br>Online: http://www.sabinet.co.za/		
[1]<br>[2]	amalgam AND composite<br>amalgam AND composite restorations	540<br>137
**Articles included**		**0**
Hand-search (Internet):		1
Reference check of included trial reports:		15
**Total articles/documents included:**		**46**

The highest search term number in brackets [] indicates utilized final search term for literature search.
Duplications of found citations are excluded.

2. Search strategy: electronic database search: HVGIC *versus* amalgam

Search term number	Electronic database	Number of Citations found
BMC search strategy: 02.12.2013 / Setting: between 2012 - 2013<br>Online: http://www.biomedcentral.com/search/		
[1]	atraumatic restorative treatment	8
**Articles included**		**0**
CENTRAL search strategy: 02.12.2013 / Setting: between 2012 – 2013/Cochrane reviews, Trials<br>Online: http://www.thecochranelibrary.com/view/0/index.html		
	#1 ART<br>#2 amalgam<br>#3 (#1 AND #2)<br>#4 in Trials<br>#5 in Trials (2012 – 2013)<br><br>#1 atraumatic restorative treatment<br>#2 in Trials<br>#3 in Trials (2012 – 2013)	131<br>8<br>0<br>0<br>0<br><br>70<br>69<br>3
**Articles included**		**0**
DOAJ search strategy: 02.12.2013 / Setting: From 2012 -2013<br>Online: http://www.doaj.org		
[1]	atraumatic restorative treatment	7
**Articles included**		**0**
GoogleScholar search strategy: 02.12.2013 / Custom range: 2012 – 2013<br>Online: http://scholar.google.co.za/		
[1]	“atraumatic restorative treatment” AND amalgam	About 142 results
**Articles included**		**0**
IndMed search strategy: 02.12.2013<br>Online: http://indmed.nic.in/		
[1]<br>[2]	ART<br>atraumatic restorative treatment	246<br>0
**Articles included**		**0**
OpenSIGLE search strategy: 02.12.2013<br>Online: http://opensigle.inist.fr/		
[1]	ART approach	98
**Articles included**		**0**
PubMed search strategy: 07.01.2012 – 02.12.2013<br>Online: http://www.pubmed.org		
[1]	atraumatic restorative treatment	44
**Articles included**		**1**
SABINET search strategy: 02.12.2013<br>Online: http://www.sabinet.co.za/		
[1]	atraumatic restorative treatment	23
**Articles included**		**0**
Hand-search (Internet):		6
Reference check of included trial reports:		0
**Total articles/documents included:**		**7**

The highest search term number in brackets [] indicates utilized final search term for literature search.
Duplications of found citations are excluded.

3. Search strategy: electronic database search: HVGIC *versus* Composite resin

Search term number	Electronic database	Number of Citations found
BMC search strategy: 02.12.2013<br>Online: http://www.biomedcentral.com/search/		
[1]	glass ionomer AND composite (All words) in All fields (full text)	25
**Articles included**		**0**
CENTRAL search strategy: 02.12.2013<br>Online: http://www.thecochranelibrary.com/view/0/index.html		
[1]	glass ionomer AND composite (Section: Trials)	400
**Articles included**		**0**
DOAJ search strategy: 02.12.2013<br>Online: http://www.doaj.org		
[1]	glass ionomer AND composite	106
**Articles included**		**0**
GoogleScholar search strategy: 02.12.2013<br>Online: http://scholar.google.co.za/		
[1]<br>[a]<br>[2]	"glass ionomer" AND composite<br>Custom range setting: 1994 – 2013<br>[1]-[a] AND "direct posterior restoration"	About 19,000 results<br>About 15,400 results<br>About 6 results
**Articles included**		**0**
IndMed search strategy: 02.12.2013<br>Online: http://indmed.nic.in/		
[1]	(glass ionomer) and (composite)	0
**Articles included**		**0**
OpenSIGLE search strategy: 02.12.2013<br>Online: http://opensigle.inist.fr/		
[1]	glass ionomer AND composite	0
**Articles included**		**0**
PubMed search strategy: 02.12.2013<br>Online: http://www.pubmed.org		
[1]-1	(("Glass Ionomer Cements"[Mesh]) AND "Composite Resins"[Mesh]) AND "Dental Restoration, Permanent"[Mesh]	1637
[1]-2	atraumatic restorative treatment	317
[1]-3	(("fuji IX" [Supplementary Concept]) OR "Fuji IX GP Fast" [Supplementary Concept]) AND "Composite Resins"[Mesh]	61
[1]-4	(((("ketac-molar" [Supplementary Concept]) OR "Ketac-Molar Quick" [Supplementary Concept]) OR "Ketac-Molar Aplicap" [Supplementary Concept]) OR "Ketac Molar Easymix" [Supplementary Concept]) AND "Composite Resins"[Mesh]	59
[1]-5	("ChemFlex" [Supplementary Concept]) AND "Composite Resins"[Mesh]	4
[1]-6	("Dental Restoration Failure"[Mesh]) AND "Glass Ionomer Cements"[Mesh]	386
[1]-7	(("Glass Ionomer Cements"[Mesh]) AND "Composite Resins"[Mesh])<br>Set limit: Clinical trial	407
**Articles included**		**0**
SABINET search strategy: 02.12.2013<br>Online: http://www.sabinet.co.za/		
[1]	glass ionomer AND composite	2
**Articles included**		**0**
Hand-search (Internet):		2
Reference check of included trial reports:		0
**Total articles/documents included:**		**2**

The highest search term number in brackets [] indicates utilized final search term for literature search.
Duplications of found citations are excluded.

Additional file S1 – 2. Internal validity criteria

1. Assessment criteria for selection bias risk

Criteria		Score				
		0	D	C	B	A
Adequate random sequence generation method reported[1]		N	Y	Y	Y	Y
Method of concealing the generated random sequence is reported that is adequate to:	Prevent its direct observation[2]	N	N	Y	Y	Y
	Prevent its correct prediction[3]	N	N	N	Y	Y
Evidence is given in some form of statistical test result that indicates the allocated random sequence was adhered to throughout the trial[4]		N	N	N	N	Y
	Conclusion	NA				A

A = Adequate randomisation; NA = Not adequate randomisation; N = Information not provided in the trial report; Y = Information provided in the trial report.

[1] The following methods are considered as inadequate: cluster randomisation, fixed block randomisation with block size 2, minimization, alternation, randomisation of teeth, use of date of birth or patient record number, “quasi”-randomisation, split-mouth, generation of the random sequence before patient recruitment.
[2] Central randomisation or sequence allocation by other than the operator(s) who apply the allocated intervention and who informs the operator(s) which (test- or control) intervention has been allocated to a particular patient only at moment of start of intervention (e.g. by phone), are considered to be adequate.
[3] Use of the Maximal randomisation procedure is considered to be adequate.
[1-3] Fulfilment of these criteria indicates adequate attempt of effective randomisation but not that the attempt was indeed successful.
[4] Any statistical test that includes the use of the Reverse Propensity Score (RPS) is considered to be adequate. Fulfilment of this criterion indicates that the attempt of effective randomisation was indeed successful, i.e. sufficient proof of low selection bias risk.

.2. - Assessment criteria for detection- and performance bias risk

Criteria	Score			
	0	C	B	A
Adequate method of masking/blinding of patients and clinicians and evaluators reported[1]	N	Y	Y	Y
No reasons for doubt discerned from the report text that masking/blinding was not successful	N	N	Y	Y
Evidence is given in some form of applied test* result that shows the masking/blinding was successful throughout the trial[2]	N	N	N	Y
Conclusion	NA			A

A = Adequate masking/blinding; NA = Not adequate masking/blinding; N = Information not provided in the trial report;

Y = Information provided in the trial report.

[1] Fulfilment of this criterion indicates adequate attempt of effective masking/blinding of effective masking/blinding but not that the attempt was indeed successful.
[2] Fulfilment of this criterion indicates that the attempt of effective masking/blinding was indeed successful, i.e. proof of lack of detection/performance bias
***** E.g. Application of a questionnaire during the trial that assesses beliefs, as to which test- or control intervention was given/received to/by a particular trial participant, followed by statistical comparison of the questionnaire results with the true allocation sequence. A non-significant result (alpha set at 5%) should sufficiently indicate successful attempt of masking/blinding and subsequent low detection- and performance bias risk.
