# Supplementary material for: Failure Rate of Direct High-Viscosity Glass-Ionomer Versus Hybrid Resin Composite Restorations in Posterior Permanent Teeth - a Systematic Review
Source: Open Dent J. 2015 Dec 22;9:438–48. doi: 10.2174/1874210601509010438 (PMC4768657; doi:10.2174/1874210601509010438)
Supplement: Supplementary file 3 [file TODENTJ-9-438_S3.pdf]

Open Access

Supplementary Material

Failure Rate of Direct High-Viscosity Glass-Ionomer *versus* Hybrid Composite Resin Restorations in Posterior Permanent Teeth - A Systematic Review of Clinical Trials

Steffen Mickenautsch* and Veerasamy Yengopal

**SYSTEM Initiative / Department of Community Dentistry, Faculty of Health Sciences, University of the Witwatersrand, 7 York Rd., Parktown/Johannesburg 2193, South Africa*

Additional file S3 – Congress abstracts

A01

6-Month Clinical Performance of a New Glass-Ionomer Restorative System
Thursday, July 15, 2010: 9 a.m. - 10:30 a.m.
Location: Rooms 120-121 (CCIB)
S. Gurgan, F.Y. Cakir, E. Firat, Z.B. Kutuk, and S. AK, Hacettepe University, Ankara, Turkey

Objective: to evaluate the 6-month clinical performance of a new packable glass-ionomer restorative system used for the restoration of Class II cavities. Methods: Twenty six patients with at least 2 but not more than 4 Class II lesions were enrolled in this study. A total of 60 lesions were randomly divided into two groups according to the restorative systems used (n=30). The lesions in Group 1, were restored with a new glass-ionomer restorative system (EQUIA/ GC) which is a combination of a packable glass-ionomer (Fuji IX GP EXTRA/ GC) and a self-adhesive nano-filled coating (G-Coat PLUS GC); whereas the lesions in Group 2 were restored with a micro-filled composite (Gradia Direct/ GC) in combination with a self-etch adhesive (G-Bond/ GC) according to the manufacturer's instructions. Two independent examiners evaluated the restorations at baseline, after 1 week and 6 months according to the modified USPHS criteria. The differences between two groups were statistically evaluated by Pearson Chi-Square test (p=0, 05). Results: At 6 month recall, 58 restorations were reviewed in 25 patients. Retention rates, anatomic form, recurrent caries, wear, surface texture, post-operative sensitivity and color match were scored as Alpha for all restorations in the two groups. For marginal adaptation, 4 restorations in Group 1 and 5 restorations in Group 2 were scored as Bravo. Besides, 1 restoration in Group 1 and 2 restorations in Group 2 were scored as Bravo for marginal discoloration. However, the differences in terms of marginal adaptation and marginal discoloration were not statistically significant (p>0, 05). Conclusions: At the end of 6 months, the clinical performance of the new glass-ionomer restorative system was as good as the micro-filled composite resin system.

A02

0649 Comparison of Glass Ionomer Cement and Composite Following ART Approach
N.K. Ersin, U. Candan, A. Aykut, O. Oncag, and C. Eronat, Ege Universitesi, Izmir, Turkey

Objectives: The aim of this study was to compare the clinical performance of a high viscosity glass ionomer cement and a packable resin-based composite following Atraumatic Restorative Treatment (ART) approach under field conditions. Methods: 276 school children aged between 7-11 years old that had bilateral matched pairs of carious posterior primary or permanent teeth were included. A split-mouth design was used in which a high viscosity glass ionomer cement, Fuji IX and a packable resin-based composite, Surefil were randomly placed on contralateral sides. The restorations were evaluated after 6 and 12 months using USPHS criteria by two experienced examiners who had not been involved in the placement of the restorations. A total of 523 ART restorations were placed in 219 children of primary teeth, 57 children of permanent teeth. Results: The 12 month cumulative survival rates of ART restorations in the primary teeth were 99 % and 98 % for glass ionomer cement and composite class I restorations, respectively while the success rates were 77 % and 92 % of class II restorations placed with the respective materials. In the permanent dentition, only class I restorations were involved and the cumulative survival rates were 100 % and 97 % for the glass ionomer cement and composite, respectively. The main failure characteristics of class II restorations were falling out and unacceptable marginal defect. The survival rates of the restorations were not influenced by an operator effect. Most of the children did not report discomfort during treatment and 73.5 % of the children were willing to receive ART restorations again. Conclusion: The clinical performance of both materials over a 12 month period was similar and the survival rates of class I ART restorations in both primary and permanent teeth were high.

A03

S. Gurgan, Dept.Restorative Dentistry, Hacettepe University, Ankara, Turkey, F. YALCIN CAKIR, Hacettepe University, Ankara, Turkey
E. FIRAT, Dept. of Restorative Dentistry, Hacettepe University, Ankara, Turkey
Z.B. Kutuk, Department of Conservative Dentistry, Hacettepe University, Faculty of Dentistry, Ankara, Turkey
S.S. Ak, Dept. of Conservative Dentistry, Hacettepe University Facuty of Dentistry, Ankara, Turkey
Y. Korkmaz, Baskent University, Ankara, Turkey

Objective: The aim of this preliminary pilot study was to evaluate the 12-month clinical performance of a new packable glass-ionomer restorative system used for the restoration of Class II cavities. Methods: Twenty six patients with at least 2 but not more than 4 Class II lesions were enrolled in this study. A total of 60 lesions were randomly divided into two groups according to the restorative systems used (n=30). The lesions in Group 1, were restored with a new glass-ionomer restorative system (EQUIA/ GC) which is a combination of a packable glass-ionomer (Fuji IX GP EXTRA/

GC) and a self-adhesive nano-filled coating (G-Coat PLUS GC); whereas the lesions in Group 2 were restored with a micro-filled composite (Gradia Direct/ GC) in combination with a self-etch adhesive (G-Bond/ GC) according to the manufacturer's instructions. Two independent examiners evaluated the restorations at baseline, after 1 week, 6 months and 12 months according to the modified USPHS criteria. The differences between two groups were statistically evaluated by Pearson Chi-Square test (p=0, 05). Results: At 12 month recall, 58 restorations were reviewed in 25 patients. Retention rates, anatomic form, recurrent caries, surface texture, post-operative sensitivity and color match were scored as Alpha for all restorations in the two groups. Two restorations in Group 1 and 2 restorations in Group 2 were scored as Bravo for marginal discoloration at 12 months recall. For marginal adaptation, 4 restorations in Group 1 and 5 restorations in Group 2 were scored as Bravo. However, the differences in terms of marginal adaptation and marginal discoloration were not statistically significant (p>0, 05). Conclusions: At the end of 12 months, the new glass-ionomer restorative system was found to be as efficacious as the micro-filled composite resin system in clinical use.

A04

0130 Survival Rate of Glass-ionomer Restorations in Small Class I Cavities

S. Poolthong, V. Pananapiradej, R. Sakoolnamarka, S. Srisawasdi, and C. Oonsombat, Chulalongkorn University, Bangkok, Thailand

Objective: To evaluate the survival rate of glass-ionomer cement as filling materials in small class I cavities of posterior permanent teeth at 1 year. Materials and Methods: Longitudinal study of the glass-ionomer fillings in permanent posterior teeth was conducted with the university's ethical approval on dental students aged 18 to 22 years. The subjects were examined for dental caries and 122 carious lesions indicated for small class I restoration of 1 mm cavity width were treated using minimal intervention approach. The glass-ionomer materials used were Fuji II LC capsule and Fuji IX capsule. Control groups were restored with Filtek Supreme/Clearfil Photo Bond and Filtek Supreme/Prime&Bond NT. The cavities were filled according to manufacturers' instructions. After one year, all restorations were evaluated on criteria of fracture behavior, secondary caries and pulpal response. Results: All the materials showed 100% alpha rate of all assessment criteria at 6 months and 1 year. Conclusion: Within 1 year, 100% of glass-ionomer restorations survive without secondary caries under function in oral cavity. Further assessment is required for longer period of services.

A05

107 24-Month Clinical Performance of a Glass-Ionomer Restorative System

Wednesday, June 20, 2012: 11 a.m. - 12:30 p.m.

Location: Foz do Iguacu Room (Mabu Hotel)

Presentation Type: Oral Session

S. Gurgan, Dept.Restorative Dentistry, Hacettepe University, Ankara, Turkey

F. Yalcin Cakir, School of Dentistry Department of Restorative Dentistry, Hacettepe University, Ankara, Turkey

E. Firat, Dept. of Restorative Dentistry, Hacettepe University, Ankara, Turkey

Z.B. Kutuk, Department of Conservative Dentistry, Hacettepe University, Faculty of Dentistry, Ankara, Turkey

S.S. Ak Oztas, Dept. of Conservative Dentistry, Hacettepe University Facuty of Dentistry, Ankara, Turkey

Y. Korkmaz Ceylan, Houston Center for Biomaterials and Biomimetics, University of Texas - Houston/Health Science Center, Houston, TX

Objective: The aim of this study was to assess the 24-month clinical performance of a glass-ionomer restorative system used for the restoration of Class II cavities. Method: Twenty six patients with at least 2 but not more than 4 Class II lesions were included in this study. A total of 60 lesions were randomly divided into two groups according to the restorative systems used (n=30). The lesions in Group 1, were restored with a glass-ionomer restorative system (EQUIA/ GC) which was a combination of a packable glass-ionomer (Fuji IX GP EXTRA/ GC) and a self-adhesive nano-filled coating (G-Coat PLUS GC); whereas the lesions in Group 2 were restored with a micro-filled composite (Gradia Direct/ GC) in combination with a self-etch adhesive (G-Bond/ GC) by two calibrated operators according to the manufacturers' instructions. Two independent examiners evaluated the restorations at baseline, 6-12-18 and 24 months according to the modified USPHS criteria. The differences between two groups were statistically evaluated by Pearson Chi-Square test (p=0. 05). Result: After 24 months, 53 restorations were evaluated in 23 patients with a recall rate of 88.3%. All the restorations in the two groups were scored as Alpha for retention rate, anatomic form, recurrent caries, surface texture, postoperative sensitivity and color match. For marginal adaptation, 4 restorations (15.4%) in Group 1 and 8 restorations (29.6%) in Group 2 were scored as Bravo. Two restorations (7.6%) in Group 1 and 5 restorations (18.5%) in Group 2 were also scored as Bravo for marginal discoloration. However, the differences in terms of marginal adaptation and marginal discoloration were not statistically significant at the end of 24 months (p>0, 05). Conclusion: The use of both materials for the restoration of Class II cavities exhibited a similar and clinically acceptable performance after 24-months.

A06

2933 36-Months Clinical Performance of a Glass-Ionomer Restorative System

Saturday, March 23, 2013: 10:45 a.m. - 12:15 p.m.

Location: Room 617 (Washington State Convention Center)

Presentation Type: Oral Session

S. Gurgan[1], F. Yalcin Cakir[1], E. Firat[1], Z.B. Kutuk[2], and S.S. Oztas[1]

[1]School of Dentistry Department of Restorative Dentistry, Hacettepe University, Ankara, Turkey

[2]Department of Restorative Dentistry, Hacettepe University, Ankara, Turkey

Objective: To evaluate the clinical performance of a glass-ionomer restorative system comparing with a microfilled resin composite used for the restoration of Class II cavities for a period of 36-months. Methods: Twenty six patients having 2 to 4 moderate size Class II lesions were included. A total of 60 lesions were randomly restored either with a glass-ionomer restorative system (EQUIA/ GC) which was a combination of a packable glass-ionomer (Fuji IX GP EXTRA/ GC) and a self-adhesive nano-filled coating (G-Coat PLUS GC) in Group1; or with a micro-filled composite (Gradia Direct/ GC) with a self-etch adhesive (G-Bond/ GC) in Group2, by two calibrated operators according to the manufacturers' instructions (n=30). Two independent examiners evaluated the restorations at baseline, 6-12-18-24 and 36 months according to the modified USPHS criteria. The differences between two groups were statistically evaluated by Pearson Chi-Square test (p=0. 05). Results: Fifty-three restorations were evaluated in 23 patients with a recall rate of 88.3%, after 36 months. All the restorations in the two groups were scored as Alpha for recurrent caries, surface texture, postoperative sensitivity and color match. For marginal adaptation, 6 restorations (23.7%) in Group 1 and 8 restorations (29.6%) in Group 2 were scored as Bravo. Two restorations (7.6%) in Group 1 and 5 restorations (18.5%) in Group 2 were scored as Bravo for marginal discoloration.

One restoration (3.8%) in Group 1 was scored as Charlie for anatomic form and retention because of marginal fracture within restorative material. However, there were no significant differences between the clinical performances of the materials at the end of 36 months for the criteria assessed ($p>0.05$). Conclusions: Both materials exhibited a similar and clinically acceptable performance on moderate Class II cavities after 36-months.
